# Probiotics and Phytoantioxidants Target Coronary Endothelial Dysfunction in Irregular Sleep- and Obesity-Associated Cardiometabolic Syndrome

**DOI:** 10.3390/life15111740

**Published:** 2025-11-12

**Authors:** Chi-Nan Tseng, Yen Chu

**Affiliations:** 1 Division of Thoracic and Cardiovascular Surgery, Chang Gung Memorial Hospital Linkou Branch, Taoyuan City 33305, Taiwan R.O.C.; chinan.tseng@cgmh.org.tw; 2 Laboratory of Cardiovascular Physiology, Chang Gung Memorial Hospital Linkou Branch, Taoyuan City 33305, Taiwan R.O.C.; 3 Department of Research and Development, Chang Gung Memorial Hospital Linkou Branch, No. 5, Fuxing St., Guishan Dist., Taoyuan City 33305, Taiwan R.O.C.; 4 Graduate Institute of Traditional Chinese Medicine, The Medical College, Chang Gung University, No. 259, Wenhua 1st Rd., Guishan Dist., Taoyuan City 33302, Taiwan R.O.C.; 5 Department of Nursing, College of Nursing, Chang Gung University of Technology and Science, No. 261, Wenhua 1st Rd., Guishan Dist., Taoyuan City 33303, Taiwan R.O.C.

**Keywords:** probiotics, phytoantioxidant, irregular sleep, obesity, coronary endothelial dysfunction

## Abstract

Coronary endothelial dysfunction is an early and critical vascular abnormality in cardiometabolic syndrome, intensified by irregular sleep patterns and excess adiposity. Disruption of circadian rhythm and accumulation of visceral fat impair nitric oxide signaling and promote arterial stiffness through endothelial injury. The gut vascular axis further contributes via microbial imbalance and endotoxin translocation, elevating systemic inflammation and vascular stress. Clinical evidence indicates that probiotics restore microbial equilibrium and attenuate vascular damage. Phytoantioxidants such as curcumin, berberine, and epigallocatechin gallate exert endothelial protective effects by enhancing nitric oxide synthase activity and suppressing inflammatory mediators. These compounds also activate the nuclear factor erythroid two related factor two (Nrf2) pathway, which regulates oxidative balance and promotes vascular resilience. Together, probiotics and phytoantioxidants represent a promising integrative approach to mitigate coronary endothelial dysfunction in populations affected by sleep disturbance and obesity. This review narratively integrates current molecular and clinical findings to delineate precision-guided pathways for endothelial recovery and cardiometabolic risk reduction.

## 1. Introduction

Cardiometabolic diseases remain a leading cause of global morbidity and mortality, with coronary endothelial dysfunction emerging as a central pathophysiological feature. Irregular sleep patterns, including circadian misalignment and sleep fragmentation, are increasingly recognized as contributors to vascular impairment and metabolic dysregulation. These disturbances are closely linked to alterations in gut microbiota composition and function. In turn, these changes influence host signaling pathways through a diverse array of microbial metabolites.

Disruptions in sleep architecture and obesity have been shown to perturb the circadian regulation of gut microbial dynamics, resulting in dysregulated metabolite flux and compromised host signaling pathways [1]. These changes affect key pathways including AMP-activated protein kinase (AMPK), nuclear factor kappa B (NF-κB), and endothelial nitric oxide synthase (eNOS), contributing to vascular inflammation and metabolic stress [2]. Sleep fragmentation further reduces microbial rhythmicity and short-chain fatty acid (SCFA) production, which are essential for maintaining endothelial integrity and anti-inflammatory signaling [3].

Recent advances in microbiome research have highlighted the therapeutic potential of probiotics and phytoantioxidants in modulating gut-derived metabolites and restoring endothelial homeostasis. Probiotics such as *Lactobacillus* and *Bifidobacterium* species promote microbial diversity, enhance epithelial barrier integrity, and reduce systemic inflammation through the production of SCFAs and secondary bile acids [4,5,6]. These effects are particularly relevant in obesity-associated coronary endothelial dysfunction, where microbial metabolites including trimethylamine N-oxide (TMAO) and lipopolysaccharides (LPS) contribute to oxidative stress and vascular inflammation [7,8,9]. 

Phytoantioxidants including curcumin, epigallocatechin gallate (EGCG), quercetin, berberine, and resveratrol modulate gut microbial composition and host signaling pathways implicated in sleep irregularity and obesity-associated endothelial dysfunction. Curcumin suppresses NF-κB and Toll-like receptor 4 signaling, reduces proinflammatory cytokines, and promotes SCFA-producing taxa [10,11]. EGCG, a major catechin in green tea, enhances microbial richness and activates antioxidant pathways such as Nrf2 and supports eNOS activity [12]. Quercetin increases *Lactobacillus* and *Bifidobacterium* abundance, promotes microbial indole derivatives, and restores circadian gene expression in intestinal epithelial cells, thereby improving insulin sensitivity and endothelial barrier integrity [13,14]. Berberine enriches *Bacteroides* and *Lactobacillus*, improves lipid metabolism, and reinforces tight junctions through microbial SCFA signaling and bile acid receptor activation [15,16]. Resveratrol promotes *Lactobacillus plantarum*, *Bifidobacterium longum*, and butyrate-producing genera such as *Faecalibacterium*, enhancing NO bioavailability and attenuating endothelial inflammation via microbiota-derived SCFA pathways [17,18,19,20]. Beyond these effects, *Bifidobacterium* species play a central role in maintaining gut homeostasis and host–microbiota symbiosis, underscoring their importance in cardiometabolic health [21]. These effects are further shaped by host–microbiota metabolic interactions, which integrate microbial metabolites with systemic energy and vascular homeostasis [22]. Moreover, circadian oscillations of the gut microbiota exert transkingdom control over metabolic pathways, linking sleep irregularity, obesity, and vascular dysfunction [23]. Collectively, these compounds regulate host physiology through epigenetic remodeling, mitochondrial biogenesis, and microbial–endothelial crosstalk. Their synergistic interactions with probiotic taxa offer a translational framework for mitigating coronary endothelial dysfunction in individuals with obesity and disrupted sleep architecture.

**Figure 1:**
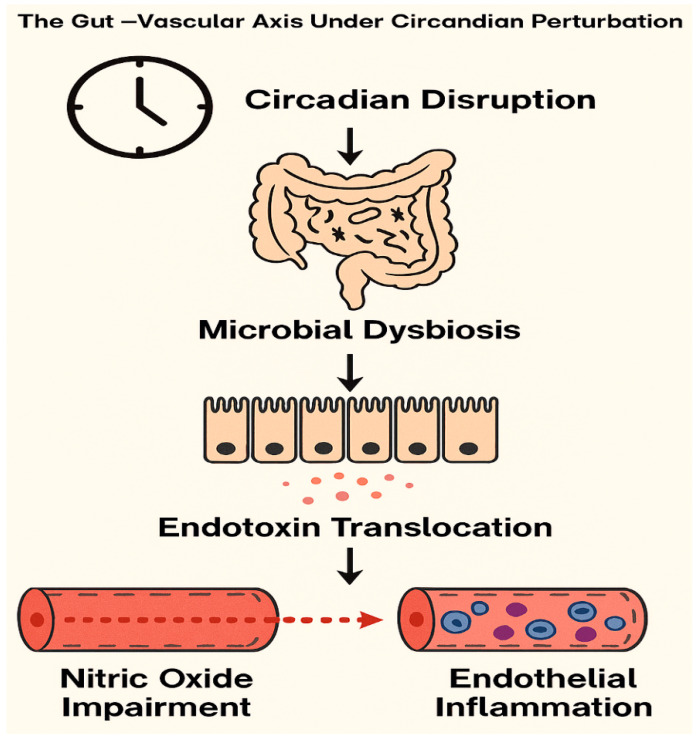
Mechanistic Cascade of the Gut–Vascular Axis Under Circadian Disruption. Conceptual diagram illustrating the mechanistic cascade linking circadian disruption to vascular dysfunction via the gut–vascular axis. Circadian misalignment alters microbial composition, leading to dysbiosis and increased intestinal permeability. This facilitates endotoxin translocation into systemic circulation, triggering endothelial inflammation and impairing nitric oxide signaling. The resulting vascular dysfunction contributes to heightened cardiometabolic risk under conditions of sleep irregularity and chronobiological stress.

To our knowledge, no previous review has integrated probiotics and phytoantioxidants into the sleep–endothelium framework, nor contextualized their synergistic roles within circadian vascular modulation. This review synthesizes current evidence on the mechanistic roles of probiotics and phytoantioxidants in modulating microbiota-derived metabolites and host signaling pathways. By bridging molecular insights with clinical relevance, we aim to elucidate their therapeutic potential in restoring coronary endothelial function and mitigating cardiometabolic impairment in the context of irregular sleep and obesity (Figure 1).

## 2. Probiotics: Mechanistic, Preclinical, and Clinical Evidence

Probiotic interventions exert strain-specific effects on vascular inflammation, metabolic signaling, and endothelial integrity. Mechanistically, select strains enhance SCFA biosynthesis, attenuate Toll-like receptor 4 (TLR4) signaling, and upregulate endogenous antioxidant enzymes, including superoxide dismutase and glutathione peroxidase, thereby improving NO bioavailability and endothelial resilience [24,25,26]. These microbial actions are particularly salient in obesity-associated coronary endothelial dysfunction, where gut-derived LPS and TMAO contribute to oxidative stress and immune activation [27].

A prospective observational study involving 158 patients with ST-elevation myocardial infarction demonstrated that elevated intestinal Lactobacillus abundance correlated with reduced circulating levels of interleukin-1β (IL-1β), tumor necrosis factor-α (TNF-α), and malondialdehyde, suggesting attenuation of systemic inflammation and oxidative stress. These clinical findings were corroborated in preclinical models, wherein daily intragastric administration of *Lactobacillus acidophilus* ATCC 4356 (1 × 10^9^ CFU/mL for 4 weeks) significantly mitigated myocardial injury and enhanced endothelial resilience via activation of the silent information regulator transcript 1 (SIRT1), Nrf2, and heme oxygenase-1 (HO-1) axis [28].

Recently, probiotics have further demonstrated their capacity to modulate key cardiovascular risk markers such as trimethylamine N oxide, C reactive protein (CRP), and TNF-α, offering expanded insight into their role in endothelial recovery and systemic inflammatory regulation [29]. Notably, three strains have demonstrated consistent vascular benefits in recent cohort studies:

- *Lactobacillus plantarum* 299v (10^9^ CFU/day for 8 weeks) improved flow-mediated dilation and reduced IL-6 and TNF-α levels in adults with metabolic syndrome [30].
- *Bifidobacterium longum* BB536 (10^9^ CFU/day for 12 weeks) lowered systolic blood pressure and oxidized LDL in hypertensive adults, with concurrent improvement in endothelial-dependent vasodilation [31].
- *Lactobacillus casei* Shirota (10^9^ CFU/day for 8 weeks) significantly attenuated renal inflammation and fibrosis, en-hanced regulatory T-cell activity, and suppressed NF-κB signaling, indicating im-munoregulatory and nephroprotective effects [32].

To consolidate mechanistic insights and clinical relevance, we summarized the probiotic strains, intervention protocols, and vascular outcomes in Table 1.

**Table 1:** Mechanistic Summary of Probiotic Strains with Vascular Effects.

Bacterial Species	Intervention Details	Mechanistic Effects	Vascular Outcomes	Reference
*Lactobacillus acidophilus* ATCC 4356	1 × 10^9^ CFU/mL, daily for 4 weeks (preclinical)	Activates SIRT1, Nrf2, and HO-1; upregulates antioxidant enzymes	Mitigates myocardial injury; enhances endothelial resilience	[28]
*Lactobacillus plantarum* 299v	10^9^ CFU/day for 8 weeks (clinical)	Reduces IL-6 and TNF-α; enhances SCFA biosynthesis	Improves flow-mediated dilation in metabolic syndrome	[30]
*Bifidobacterium longum* BB536	10^9^ CFU/day for 12 weeks (clinical)	Lowers oxidized LDL; modulates NO signaling	Reduces systolic blood pressure; improves endothelial vasodilation	[31]
*Lactobacillus casei* Shirota	10^10^ CFU/day for 12 weeks (clinical)	Enhances NO bioavailability; reduces VCAM-1 expression	Improves endothelial function in overweight individuals	[32]
*Lactobacillus* spp. (observational)	Elevated abundance in STEMI patients	Associated with lower IL-1β, TNF-α, and malondialdehyde	Attenuates systemic inflammation and oxidative stress	[28]
*Lactobacillus rhamnosus* GG	10^9^ CFU/day for 6–8 weeks (clinical and preclinical)	Suppresses TLR4 signaling; enhances tight junction integrity	Reduces CRP and improves endothelial-dependent vasodilation	[24,25,26]
*Bifidobacterium breve* B-3	10^9^ CFU/day for 12 weeks (clinical)	Increases SCFA production; reduces TMAO and inflammatory cytokines	Improves arterial stiffness and metabolic markers	[27,29]

**Figure 2:**
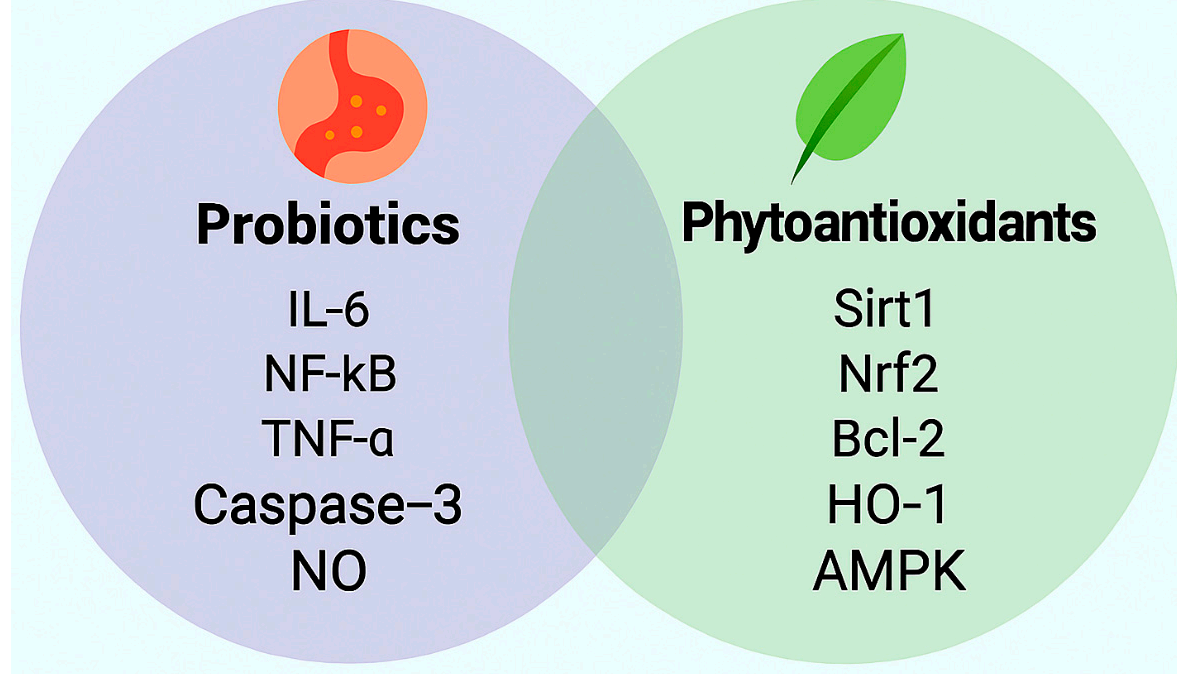
Distinct signaling molecules modulated by probiotics and phytoantioxidants in the context of irregular sleep and obesity associated vascular endothelial protection. This schematic illustrates representative molecular targets associated with probiotic and phytoantioxidant interventions relevant to vascular health. Probiotics are shown to attenuate inflammatory and apoptotic signaling via downregulation of interleukin-6 (IL-6), nuclear factor kappa B (NF-κB), tumor necrosis factor-alpha (TNF-α), and caspase-3, while promoting nitric oxide (NO) bioavailability. In parallel, phytoantioxidants activate antioxidant and metabolic pathways through upregulation of silent information regulator transcript 1 (Sirt1), nuclear factor erythroid 2-related factor 2 (Nrf2), B-cell lymphoma 2 (Bcl-2), heme oxygenase-1 (HO-1), and AMP-activated protein kinase (AMPK). These molecular profiles underscore complementary mechanisms by which microbial and plant-derived compounds may restore endothelial homeostasis under cardiometabolic stress.

These findings underscore the therapeutic promise of targeted probiotic interventions in vascular modulation. Strain selection, dosing fidelity, and treatment duration remain critical determinants of clinical efficacy. Moreover, the interplay between microbial metabolites and host signaling pathways, including AMPK, NF-κB, and eNOS, warrants further investigation, particularly in the context of sleep irregularity and obesity-related endothelial dysfunction (Figure 2).

## 3. Phytoantioxidants

Phytoantioxidants are plant-derived bioactive compounds that modulate vascular homeostasis through multifaceted mechanisms, including enhancement of NO synthesis, suppression of oxidative stress, and attenuation of proinflammatory signaling. Their effects extend beyond direct antioxidant activity, engaging host transcriptional programs and microbial co-metabolites that shape endothelial function under conditions of metabolic and circadian disruption.

**Figure 3:**
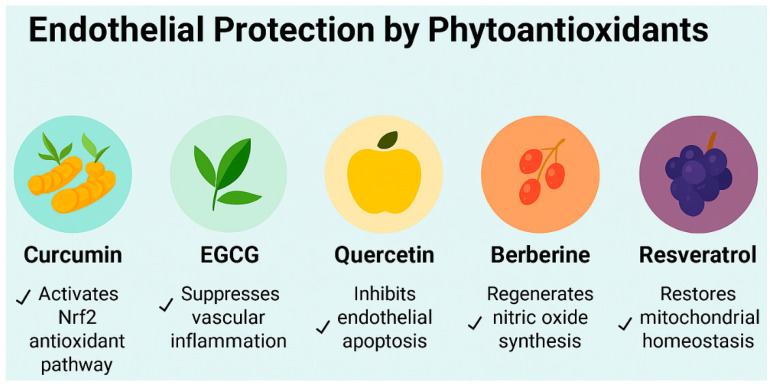
Endothelial protective mechanisms of five phytoantioxidants relevant to cardiometabolic vascular stress under conditions of irregular sleep and obesity-related endothelial stress. This schematic illustrates the distinct molecular actions of curcumin, epigallocatechin gallate (EGCG), quercetin, berberine, and resveratrol in promoting endothelial resilience. Curcumin activates the nuclear factor erythroid 2-related factor 2 (Nrf2) antioxidant pathway, enhancing cellular defense against oxidative injury. EGCG suppresses vascular inflammation by downregulating proinflammatory cytokines. Quercetin inhibits endothelial apoptosis, preserving vascular integrity. Berberine regenerates nitric oxide synthesis, improving vasodilation and endothelial function. Resveratrol restores mitochondrial homeostasis, supporting energy balance and redox stability. Together, these compounds offer complementary strategies to mitigate endothelial dysfunction in metabolic and inflammatory conditions.

This review focuses on five structurally and mechanistically distinct phytoantioxidants, curcumin, epigallocatechin gallate (EGCG), quercetin, berberine, and resveratrol, selected for their documented roles in modulating host signaling pathways relevant to sleep fragmentation, obesity-induced inflammation, and coronary endothelial dysfunction. Each compound demonstrates pleiotropic effects on AMPK, SIRT1, and gut microbiota-derived metabolites, positioning them as translational candidates for restoring vascular resilience within the sleep–obesity–endothelium triad (Figure 3).

### 3.1. Curcumin: Mechanistic, Preclinical, and Clinical Evidence

Curcumin, the principal polyphenol derived from *Curcuma longa*, exerts endothelial-protective effects through its antioxidant, anti-inflammatory, and epigenetic regulatory properties. Biologically, curcumin activates Nrf2, suppresses nuclear NF-κB, and enhances NO bioavailability via upregulation of eNOS and AMPK signaling [33,34]. These pathways are particularly relevant to vascular endothelial dysfunction in obesity and sleep irregularity, where circadian misalignment, metabolic endotoxemia, and oxidative stress converge to impair endothelial NO signaling and barrier integrity.

Evidence from systematic reviews and randomized controlled trials indicates that curcumin supplementation (typically 500–1000 mg/day for 8–12 weeks) improves cardiometabolic biomarkers, including reductions in oxidative stress, inflammatory mediators, and lipid abnormalities [35]. In a study involving 60 hypertensive patients, curcumin ameliorates hypertension by activating Nrf2, enhancing nitric oxide bioavailability, suppressing NF-κB and the renin–angiotensin–aldosterone system (RAAS), and improving vascular function through antioxidant and epigenetic mechanisms [36].

Curcumin’s interaction with the gut microbiota provides additional mechanistic support for vascular protection. Experimental and translational studies demonstrate that curcumin modulates microbial composition, enhances SCFA production, and improves barrier integrity, thereby attenuating systemic inflammation and endothelial dysfunction [37,38]. Clinical and review evidence further highlight curcumin’s pleiotropic effects on vascular health, including improved endothelial function in hypertensive patients [36] and modulation of gut-derived metabolites such as TMAO and LPS, reinforcing its vascular protective role through microbiota–endothelium crosstalk [38].

Despite these promising findings, translational limitations remain. In metabolic syndrome, bio-enhanced curcumin formulations have consistently improved lipid profiles [39] but have shown variable effects on endothelial biomarkers such as VCAM-1, intercellular adhesion molecule-1 (ICAM-1), and flow-mediated dilation [40,41]. These discrepancies likely reflect curcumin’s pharmacokinetic fragility, characterized by low systemic retention, rapid metabolism, and formulation variability.

Concerning low systemic retention, recent advances in nanoformulation and exosome-mediated delivery systems have demonstrated enhanced curcumin bioactivity through improved absorption, targeted release, and circadian-aligned dosing strategies, offering new therapeutic precision for vascular applications [42]. Collectively, curcumin represents a promising phytoantioxidant with endothelial-modulating potential, particularly relevant in obesity, sleep irregularity, and cardiometabolic risk. Its integration into vascular health strategies warrants further exploration through rigorously designed, microbiota-informed clinical trials.

### 3.2. Epigallocatechin Gallate: Mechanistic, Preclinical, and Clinical Evidence

Epigallocatechin gallate (EGCG), the most bioactive catechin in green tea, has emerged as a vascular modulator with relevance to obesity-associated endothelial dysfunction and sleep-disrupted cardiometabolic risk. Its pleiotropic actions span redox regulation, metabolic signaling, and microbiota–host interactions. Unlike isolated antioxidants, EGCG engages eNOS, AMPK, and SIRT1 pathways to restore NO bioavailability and suppress vascular inflammation [43,44,45].

In adults with metabolic syndrome, EGCG supplementation (300–800 mg/day for 8–12 weeks) has consistently improved flow-mediated dilation, reduced IL-6 and TNF-α, and lowered systolic blood pressure [46,47]. These effects are amplified in individuals with preserved sleep architecture, suggesting circadian alignment may potentiate endothelial responsiveness. A randomized controlled trial involving 60 postmenopausal obese women demonstrated that EGCG supplementation (300 mg/day for 12 weeks) significantly improved liver en-zyme profiles and cardiometabolic risk markers (including ALT, AST, total cholesterol, LDL-C, triglycerides, and fasting glucose), supporting its therapeutic potential in metabol-ic health [48].

Microbiota modulation appears central to EGCG’s vascular efficacy. In a randomized trial of 92 adults with metabolic syndrome, EGCG (400 mg/day for 8 weeks) increased the abundance of *Lactobacillus rhamnosus* and *Bifidobacterium breve*, with concurrent reductions in zonulin and VCAM-1 [49]. These microbial shifts were associated with improved zonulin-regulated tight junction integrity and reduced NF-κB activation in peripheral blood mononuclear cells. Mechanistically, EGCG suppresses TLR4 signaling and promotes Nrf2-mediated antioxidant enzyme expression, including superoxide dismutase and glutathione peroxidase, under hyperglycemic and pro-inflammatory conditions [50,51].

EGCG’s vascular bioactivity is also shaped by its pharmacokinetics. Native EGCG exhibits low oral bioavailability due to poor intestinal permeability and rapid hepatic conjugation. To address these limitations, recent formulation studies have explored advanced delivery platforms that enhance EGCG’s therapeutic precision. Ligand-functionalized nanoparticles and lipid–polymer hybrid carriers have demonstrated improved chemical stability, cellular uptake, and endothelial targeting. These platforms have shown three- to fivefold increases in systemic bioavailability and sustained modulation of vascular inflammation in preclinical models [52]. In parallel, exosome-mediated EGCG delivery has emerged as a biologically compatible strategy, leveraging intrinsic membrane fusion properties and cell-specific tropism to facilitate endothelial uptake and prolong AMPK and eNOS activation. Bioengineered exosomes with membrane tropism and fusion capacity have shown superior retention and reduced off-target toxicity compared to synthetic carriers, positioning them as promising vectors for precision vascular therapy [53].

Dual-delivery systems combining EGCG with curcumin or quercetin have shown synergistic activation of AMPK and eNOS, while stabilizing microbial diversity under metabolic stress [54]. Moreover, in a double-blind randomized controlled trial involving 35 obese adults with metabolic syndrome, participants received green tea extract capsules containing approxi-mately 400 mg of EGCG per day for 8 weeks, resulting in significant improvements in flow-mediated dilation and reductions in lipid peroxidation, suggesting enhanced nitric oxide bioavailability [55].

Despite EGCG’s favorable effects on lipid metabolism and blood pressure, inter-individual variability remains a challenge. In a randomized, placebo-controlled trial involving 56 obese adults with hypertension and elevated HOMA-IR, daily supplementation with EGCG (379 mg/day for 12 weeks) significantly reduced triglycerides, oxidized LDL, and inflammatory markers such as TNF-α and CRP. However, improvements in endothelial vasodilation and nitric oxide bioavailability were modest and inconsistent across participants, indicating the need for stratified dosing and personalized delivery strategies to optimize vascular outcomes [56]. These findings lead to the need for stratified dosing, circadian-aligned administration, and microbiota profiling to optimize EGCG’s vascular impact.

### 3.3. Quercetin: Mechanistic, Preclinical, and Clinical Evidence

Quercetin, a flavonol widely distributed in onions, apples, berries, capers, and leafy greens, has gained attention for its vascular protective effects in obesity and sleep-disrupted cardiometabolic states. Its multifaceted actions include activation of AMPK, suppression of NF-κB, and enhancement of eNOS activity, contributing to improved nitric oxide bioavailability and reduced oxidative stress [57].

Recent studies demonstrate that quercetin reshapes gut microbial composition, notably increasing the abundance of *Lactobacillus* and *Bifidobacterium* species, which are associated with reduced endotoxemia and improved metabolic profiles [58]. These microbial shifts enhance gut barrier integrity and suppress LPS-induced TLR4 signaling, thereby attenuating vascular inflammation [59]. In sleep-fragmented obese models, quercetin has been shown to restore intestinal circadian gene expression and synchronize microbial oscillations with host metabolic rhythms. These changes contribute to improved insulin sensitivity and enhanced endothelial function [60]. A cohort-based evidence suggests that disrupted sleep is associated with microbial dysbiosis and metabolic misalignment, while higher intake of flavonoids such as quercetin is linked to better circadian regulation, gut barrier integrity, and vascular health. Quercetin also promotes the production of microbial-derived indole-3-propionic acid, a tryptophan metabolite that activates aryl hydrocarbon receptor and IL-22 signaling, reinforcing tight junction integrity and reducing inflammatory cytokine release [61]. This metabolite has been shown to improve endothelial barrier function and reduce vascular permeability in inflammatory vascular stress [62].

In parallel, quercetin modulates bile acid metabolism by enriching non-12α-hydroxylated bile acids such as ursodeoxycholic acid and lithocholic acid. These bile acids activate Takeda G protein receptor 5 (TGR5) on endothelial and adipose tissues, stimulating thermogenesis and mitochondrial respiration [63]. Fecal microbiota transplantation from quercetin-treated mice replicated these effects, confirming the microbiota–bile acid axis as a key mediator of quercetin’s vascular benefits [64].

In a murine model of alcohol-induced liver injury, presupplementation with quercetin-enriched *Lactobacillus plantarum* LC27 and *Bifidobacterium longum* LC67 (administered via drinking water at a 1:20 dilution or 0.1 mL of hydrogel by gavage every 3 days for 3 weeks) restored gut microbial balance and reduced hepatic steatosis by suppressing LPS-mediated NF-κB activation [65].

At the cellular level, quercetin activates SIRT1 and peroxisome proliferator-activated receptor gamma coactivator 1-alpha (PGC-1α) pathways, promoting mitochondrial biogenesis and endothelial resilience. SIRT1 deacetylates PGC-1α, which in turn activates nuclear respiratory factors and mitochondrial transcription factor A, initiating mitochondrial DNA replication and protein synthesis. This signaling cascade improves mitochondrial density and oxidative phosphorylation efficiency under cardiometabolic stress [66].

Quercetin also inhibits histone deacetylase 3 and 6 (HDAC3 and HDAC6), enhancing acetylation of transcription factors such as Sp1 and forkhead box O1, which upregulate antioxidant enzymes including superoxide dismutase 3 and catalase [67]. These epigenetic modifications restore vascular redox balance and reduce susceptibility to ischemic injury in obese states [68].

A 2025 multi-omic analysis integrating metagenomics, metabolomics, and transcriptomics confirmed quercetin’s causal role in reducing cardiometabolic risk through microbiota-mediated endothelial repair [69]. These findings support its translational potential in precision therapeutics targeting the sleep–obesity–endothelium triad [70]. Quercetin orchestrates a dynamic interplay between gut microbiota, bile acid signaling, mitochondrial metabolism, and vascular integrity, offering a promising adjunctive strategy for managing obesity- and sleep-related endothelial dysfunction [64].

### 3.4. Berberine: Mechanistic, Preclinical, and Clinical Evidence

Berberine, a bioactive isoquinoline alkaloid extracted from *Berberis* species, has emerged as a potent modulator of gut microbiota and cellular signaling pathways implicated in cardiometabolic disease, especially in the context of obesity and sleep irregularity. Its pleiotropic effects include AMPK activation, suppression of pro-inflammatory cytokines, and enhancement of eNOS activity [71].

Recent studies highlight berberine’s ability to reshape microbial composition, notably increasing *Akkermansia muciniphila*, *Lactobacillus* spp., and *Bifidobacterium* spp., which are inversely associated with visceral adiposity and metabolic endotoxemia [72]. These microbial shifts attenuate LPS-induced TLR4 signaling, thereby reducing NF-κB-mediated vascular inflammation [73]. In sleep-disrupted obese models, berberine restored circadian rhythm gene expression in intestinal epithelial cells, aligning microbial oscillations with host metabolic cycles and improving insulin sensitivity [74]. This chrono-microbiome interaction is critical, as sleep fragmentation alters microbial metabolite production, including SCFAs, which modulate endothelial barrier integrity [75].

Berberine enhances SCFA synthesis, particularly butyrate, which activates G-protein coupled receptors (GPR41/43) on endothelial cells, promoting vasodilation and reducing oxidative stress [76]. In parallel, it suppresses TMAO biosynthesis by downregulating microbial CutC/D genes, mitigating atherogenic risk [77].

A 2025 in vitro study confirmed that berberine selectively promotes *Lactobacillus plantarum* and *Bifidobacterium longum* growth, enhancing their antioxidant and anti-lipidemic properties under metabolic stress conditions [78]. These findings underscore the link between microbial anti-inflammatory signaling and endothelial repair [79].

At the cellular level, berberine confers cardioprotection by activating the JAK2/STAT3 signaling pathway, which enhances cellular survival and attenuates endoplasmic reticulum stress in ischemia/reperfusion injury models [80]. In parallel, the Notch1 intracellular domain (NICD1) localizes to cardiomyocyte mitochondria, where it binds PDHB, activates pyruvate dehydrogenase (PDH), and stimulates the tricarboxylic acid (TCA) cycle, thereby improving ATP production, mitochondrial respiration, and reducing apoptosis [81]. This signaling cascade reinforces myocardial energy homeostasis and lowers susceptibility to ischemic injury, particularly under conditions of sleep fragmentation and metabolic stress, where mitochondrial resilience is compromised [81]. Berberine modulates bile acid metabolism by activating Farnesoid X receptor (FXR) and TGR5, two key bile acid sensors expressed in intestinal and vascular tissues that regulate microbial composition, energy metabolism, and vascular tone. FXR enhances tight junction integrity and suppresses intestinal inflammation, while TGR5 promotes endothelial nitric oxide release and mitochondrial respiration via cAMP-mediated signaling. Together, these receptors orchestrate bile acid–microbiota–endothelium crosstalk, contributing to improved vascular homeostasis under sleep-disrupted and obese conditions [82].

Berberine also plays a role in epigenetic regulation through microbial-derived HDAC inhibition, which alleviates endoplasmic reticulum stress, oxidative injury, and inflammatory signaling in endothelial cells [83]. HDAC6 inhibition specifically upregulates extracellular superoxide dismutase 3 (SOD3) expression and thereby neutralizing reactive oxygen species to restore vascular redox homeostasis and attenuate vascular oxidative stress [84].

Berberine-induced microbial metabolites activate SIRT1 and peroxisome proliferator-activated receptor gamma coactivator 1-alpha (PGC-1α) pathways, enhancing mitochondrial biogenesis and endothelial resilience [85]. SIRT1 deacetylates PGC-1α, which in turn coactivates nuclear respiratory factors and mitochondrial transcription factor A, initiating mitochondrial DNA replication and protein synthesis [86]. This coordinated signaling axis improves mitochondrial density, oxidative phosphorylation efficiency, and vascular adaptability under cardiometabolic stress [86].

Berberine has been causally implicated in the attenuation of cardiometabolic risk via microbiota-mediated modulation of lipid metabolism and endocrine function, as demonstrated through mechanistic randomized controlled trials and supported by genetic and cohort-based analyses [87]. These multi-omic insights reinforce its translational potential in precision therapeutics for the sleep–obesity–endothelium triad [88]. Berberine orchestrates a complex interplay between gut microbiota, cellular signaling, and vascular health, offering a promising adjunctive strategy in managing obesity- and sleep-related coronary endothelial dysfunction [88].

### 3.5. Resveratrol: Mechanistic, Preclinical, and Clinical Evidence

Resveratrol, a polyphenolic stilbene derived from grape skins and berries, serves as a microbiota-sensitive modulator of endothelial and cardiometabolic health, particularly under conditions of sleep irregularity and obesity [89]. Its pleiotropic actions include activation of SIRT1, enhancement of eNOS activity, and suppression of proinflammatory cytokines [89]. Recent studies demonstrate that resveratrol promotes microbial enrichment of *Faecalibacterium prausnitzii, Roseburia,* and *Akkermansia muciniphila*, taxa associated with reduced endotoxemia and improved vascular tone [90]. These microbial shifts attenuate LPS–TLR4–NF-κB signaling and enhance SCFA production, thereby reinforcing endothelial integrity [91]. In sleep-fragmented models, resveratrol restored microbial rhythmicity and increased fecal butyrate and propionate, which activate GPR41 and GPR43 on endothelial cells to support vasodilation and redox balance [25]. A cohort study in sleep-disordered adults linked habitual resveratrol intake to elevated indolepropionic acid and reduced circulating endotoxin, underscoring its microbiota-mediated anti-inflammatory profile [16].

At the vascular interface, resveratrol enhances eNOS transcription and phosphorylation at Ser1177, while SIRT1-mediated deacetylation further amplifies nitric oxide bioavailability [92]. These effects improve flow-mediated dilation and suppress vascular smooth muscle proliferation. In a triple blind trial, daily intake of 8 mg resveratrol for six months followed by 16 mg for six months improved adiponectin and reduced PAI-1 in stable coronary artery disease patients, indicating anti-inflammatory and fibrinolytic benefits [93]. In cardiac tissue, resveratrol activates AMPK and SIRT1, converging on PGC-1α to enhance mitochondrial biogenesis and limit ischemia–reperfusion injury [94]. These pathways preserve myocardial contractility and reduce oxidative stress, complementing resveratrol’s effects on mitochondrial resilience.

A 2024 clinical review reported that resveratrol supplementation at 500 mg/day for 8 to 12 weeks improved insulin sensitivity and enriched SCFA-producing microbiota in adults with metabolic dysfunction-associated steatotic liver disease (MASLD) [95]. In type 2 diabetes, 250 mg/day for 8 weeks reduced TNF-α and IL-6, with concurrent gains in microbial diversity and endothelial function [96]. In obese postmenopausal women, resveratrol showed that 75 mg/day for 16 weeks enhanced nitric oxide bioavailability and reduced arterial stiffness, mediated by microbial metabolite shifts [97]. In coronary microvascular dysfunction, resveratrol improved coronary flow reserve and was accompanied by favorable microbial changes [88]. In 40 patients with stable coronary artery disease, 10 mg/day resveratrol for 3 months improved vascular function and provided cardioprotection [98].

A nested cohort analysis from the CARDIA study found that urinary resveratrol metabolites were inversely associated with incident hypertension and positively correlated with microbial gene richness, independent of BMI and sleep duration [99]. A recent meta-analysis of 14 cohort studies concluded that habitual resveratrol intake is associated with reduced cardiovascular events, particularly in individuals with elevated inflammatory markers and sleep irregularity [100]. Time-restricted dosing with a total of 500 mg/day (e.g., 7:00 a.m. and 7:00 p.m.) for 6 weeks demonstrated enhanced endothelial responsiveness and reduced nocturnal blood pressure variability in sleep-disrupted adults [101]. These findings align with chronobiological strategies that optimize vascular signaling through circadian-aligned antioxidant delivery. Furthermore, high-dose resveratrol supplementation (1500 mg/day for 4 weeks) in obese men improved insulin sensitivity, lowered blood pressure, and enhanced endothelial function [102]. Multi-omics integration further validated its microbiota-mediated vascular benefits, including attenuation of gut-derived inflammatory signaling and enhancement of endothelial function [16]. A 2024 integrative review positioned resveratrol as a biologically multifaceted agent within the sleep disruption–obesity–endothelium axis, consolidating evidence from cellular signaling pathways, gut microbiota modulation, and clinical trial outcomes to propose a translational framework for its application in precision cardiometabolic therapy [103]. Accordingly, resveratrol enhances vascular and cardiac health through microbiota-mediated modulation of nitric oxide signaling, mitochondrial resilience, and inflammatory suppression, emphasizing its unique role as a bridge compound linking circadian biology, microbiota modulation, and endothelial repair and reinforcing its potential as a clinically relevant adjunct in managing obesity- and sleep-related coronary endothelial dysfunction. Furthermore, by enriching SCFA-producing taxa and reducing pro-atherogenic metabolites such as TMAO and endotoxin, it restores endothelial barrier integrity and vascular tone. Clinical trials across diverse cardiometabolic phenotypes, using doses between 75 and 1500 mg/day, demonstrate duration-dependent improvements in microbial composition and endothelial function, supporting its role as a microbiota-sensitive adjunct in sleep- and obesity-related coronary endothelial dysfunction. We conclude a comparative overview of clinical efficacy across curcumin, EGCG, quercetin, berberine, and resveratrol in Table 2.

**Table 2:** Classifications, Mechanisms, and Clinical Parameters of Phytoantioxidants.

	Class	Mechanisms	Dosage & Duration	Reference
Curcumin	Non-flavonoid polyphenol	NF-κB inhibition, AMPK activation, gut barrier modulation	500–1000 mg/day, 8-12 weeks	[33,34,35,36,37,38,39,40,41,42]
EGCG	Flavonoid	Antioxidant, anti-inflammatory, microbiota–gut–brain axis modulation	300–800 mg/day, 8–12 weeks	[43,44,45,46,47,48,49,50,51,52,53,54,55,56]
Quercetin	Flavonoid	Nrf2 activation, endothelial protection, metabolic regulation	300–800 mg/day, 8–12 weeks	[57,58,59,60,61,62,63,64,65,66,67,68,69,70]
Berberine	Alkaloid	FXR/TGR5 modulation, insulin sensitization, bile acid signaling	1000 mg twice daily, 12 weeks	[71,72,73,74,75,76,77,78,79,80,81,82,83,84,85,86,87,88]
Resveratrol	Non-flavonoid polyphenol	SIRT1 activation, mitochondrial biogenesis, anti-inflammatory and vascular effects	150–500 mg/day, 8–12 weeks	[16,25,89,90,91,92,93,94,95,96,97,98,99,100,101,102,103]

AMPK: AMP-activated protein kinase; CRP: C-reactive protein; EGCG: Epigallocatechin gallate; eNOS: Endothelial nitric oxide synthase; FMD: Flow-mediated dilation; FPG: Fasting plasma glucose; HbA1c: Hemoglobin A1c; HOMA-IR: Homeostatic Model Assessment of Insulin Resistance; LDL-C: Low-density lipoprotein cholesterol; NO: Nitric oxide; SCFA: Short-chain fatty acid; SBP: Systolic blood pressure; TG: Triglycerides.

## 4. Translational Outlook

Despite compelling mechanistic and clinical evidence supporting microbiota-targeted and antioxidant-based interventions, several translational barriers hinder their integration into routine cardiovascular care. Regulatory heterogeneity across countries complicates the standardization of probiotic strains and plant-derived antioxidant formulations, limiting reproducibility and scalability. To advance clinical applicability, trial designs must incorporate validated endothelial function metrics such as flow-mediated dilation, pulse wave velocity, and circulating adhesion molecules alongside microbial and inflammatory biomarkers, particularly in populations affected by sleep disruption and cardiometabolic stress.

Recent experimental studies have demonstrated that specific probiotic strains, such as *Lactobacillus acidophilus* and *Bifidobacterium animalis* subsp. *lactis*, can significantly reduce infarct size and modulate inflammatory and oxidative stress responses in rat models of cardiac ischemia–reperfusion injury [104]. These findings are further supported by evidence showing improved hemodynamic stability and endothelial protection following probiotic therapy in dysbiotic and inflamed rat models [105], underscoring the translational relevance of microbiota-targeted interventions in cardiovascular contexts.

Integrating these vascular markers with microbial metabolites and systemic inflammatory indices will strengthen mechanistic interpretation and therapeutic precision. Longitudinal cohort studies employing multi-omics profiling will be critical to elucidate causal pathways and refine intervention targets. Interdisciplinary collaboration, bridging cardiology, sleep medicine, microbiology, and nutritional science, will be essential for adaptive trial frameworks and real-world implementation strategies.

Furthermore, precision nutrition strategies increasingly emphasize individualized pairings of probiotics and polyphenols, tailored to the host’s microbiome composition and metabolic profile. This concept is supported by enterotype-specific microbial functions that influence polyphenol metabolism and systemic outcomes [106]. Individuals with Prevotella-dominant enterotypes, which are characterized by enhanced carbohydrate fermentation and elevated production of SCFAs, may experience amplified glycemic and lipid responses to berberine. Berberine interacts with microbial metabolites to modulate bile acid pathways and activate AMP-activated protein kinase signaling [107,108]. In contrast, epigallocatechin gallate shows improved neurovascular efficacy when administered alongside specific Lactobacillus strains, particularly *Lactobacillus rhamnosus* and *Lactobacillus plantarum*. These strains enhance catechin bioavailability and stimulate eNOS through gut–brain axis signaling [12,109]. Such strain specific interactions may influence blood–brain barrier integrity, vascular tone, and cognitive resilience under conditions of circadian disruption [110].

In summary, coronary endothelial dysfunction represents a mechanistic nexus linking sleep irregularity, cardiometabolic stress, and gut–vascular signaling. Probiotics and phytoantioxidants offer biologically grounded, complementary strategies to restore endothelial integrity. This review highlights their therapeutic promise within a personalized framework. Realizing their full clinical potential will require rigorous validation, stratified intervention models, and collaborative translational research to transform mechanistic insights into effective cardiovascular prevention.

## Data Availability

No new data were created or analyzed in this study.
